# Mechanical Field Guiding Structure Design Strategy for Meta‐Fiber Reinforced Hydrogel Composites by Deep Learning

**DOI:** 10.1002/advs.202310141

**Published:** 2024-03-23

**Authors:** Chuanzhi Liu, Xingyu Zhang, Xia Liu, Qingsheng Yang

**Affiliations:** ^1^ School of Mathematics Statistics and Mechanics Beijing University of Technology Beijing 100124 China

**Keywords:** deep learning, inverse structure design, meta‐fiber, meta‐fiber reinforced hydrogel, targeted mechanical field

## Abstract

Fiber‐reinforced hydrogel composites are widely employed in many engineering applications, such as drug release, and flexible electronics, with more flexible mechanical properties than pure hydrogel materials. Comparing to the hydrogel strengthened by continuous fiber, the meta‐fiber reinforced hydrogel provides stronger individualized design ability of deformation patterns and tunable stiffness, especially for the elaborate applications in joint, cartilage, and organ. In this paper, a novel structure design strategy based on deep learning algorithm is proposed for hydrogel reinforced by meta‐fiber to achieve targeted mechanical properties, such as stress and displacement fields. A solid mechanic model for meta‐fiber reinforced hydrogel is first developed to construct the dataset of fiber distribution and the corresponding mechanical properties of the composite. Generative adversarial network (GAN) is then trained to characterize the relationship between stress or displacement field, and meta‐fiber distribution. The well‐trained GAN is implemented to design meta‐fiber reinforced hydrogel composite structure under specific operation conditions. The results show that the deep learning method may efficiently predict the structure of the hydrogel composite with satisfied confidence, and has great potential for applications in drug delivery and flexible electronics.

## Graphical abstract

A generative structure design strategy is proposed for meta‐fiber reinforced hydrogel composite by generative adversarial network, to achieve targeted stress and displacement fields. The proposed methodology provides an efficient way for determination of the structure of complex composite structure, especially for the composite with open deign horizontal and infinite design freedom.

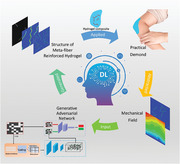

## 1 Introduction

Hydrogel receives highly scientific attention in soft robotics,^[^ ^1^, ^2^ ^]^ biomedical‐engineering,^[^ ^3^, ^4^, ^5^ ^]^ and flexible electronics engineering,^[^ ^6^, ^7^ ^]^ due to its high recoverability, good biocompatibility, and sensitivity to environmental stimulus. However, traditional hydrogels commonly show poor mechanical strength and toughness and have limits in engineering applications.^[^ ^8^, ^9^, ^10^ ^]^ As a way of improving the mechanical properties of traditional hydrogel, fiber‐reinforcement hydrogel composites possess high strength, good toughness, high fatigue threshold, and rapid recovery,^[^ ^11^, ^12^ ^]^ and more importantly, to obtain anisotropy^[^ ^13^ ^]^ in mechanical field. Hence, a series of constitutive models are proposed for describing the anisotropic deformation of fiber‐reinforced hydrogel during the swelling process. For instance, a unified intrinsic model for anisotropic fiber‐reinforced hydrogel was derived by considering the effect of fiber swelling in the Flory‐Rehner theory.^[^ ^14^ ^]^ An anisotropic constitutive model was deduced by kinematic constraint between the mechanical and chemical fields through the second law of thermodynamics.^[^ ^15^ ^]^ Shruti et al. studied the anisotropic elastic behavior for replacement heart valves.^[^ ^16^ ^]^ Moreover, the chemo‐mechanical coupling model was developed by a general form of free energy function considering chain entanglements.^[^ ^17^ ^]^ The fibers in hydrogel composite could either be segmental or continuous fibers, in which the segmental fibers, namely short fibers, may bring larger design flexibility on anisotropy. As one type of the segmental fibers, the meta‐fiber first experiences structural deformation and then material deformation during the tensile process,^[^ ^18^ ^]^ due to the geometric feature, producing exotic mechanical properties such as multi‐direction deformation, tunable stiffness, negative compressibility, and negative Poisson's ratio.^[^ ^19^, ^20^, ^21^ ^]^ Therefore, meta‐fiber reinforced hydrogel may enhance the overall mechanical properties by a reduction in stress concentration after deformation, which provides a wider design space for a tunable mechanical property of composites.

Additionally, the optimal design of a hydrogel composite usually relies on the quantitative analysis of the mechanical field, such as stress and displacement fields, to avoid mechanical failure and disability.^[^ ^22^, ^23^, ^24^ ^]^ For example, the stress distribution was designed for the optimized structure of hydrogel‐based actuators, such as finger‐like grippers, soft robots, and artificial scaffolds.^[^ ^25^, ^26^, ^27^ ^]^ The displacement field was used to characterize the complex shape transformation,^[^ ^28^ ^]^ for numerically studying the dehydration progress of leaves. The traditional structure design strategies, such as topology optimization and trial‐and‐error, require high computational penalty and longtime consuming. Therefore, an efficient strategy is necessary for the optimal structure design of meta‐fiber reinforced hydrogel composites with targeted macroscopic properties.

As an alternative way, the Deep learning (DL) method provides a perspective to explore the mechanical properties of composite and inversely design the composite structure or provide a design strategy to help researchers predict the field‐dependent parameters for the characterization and design of smart material,^[^ ^29^, ^30^, ^31^ ^]^ via learning large data from the simulation or experiments. Moreover, it benefits of solving time‐consuming and insufficient computational ability problems in complicated tasks, compared with finite element method and molecular dynamics simulation. Many types of DL methods have been employed in solid mechanics, such as U‐Net neural‐network, convolutional neural network (CNN), and generative adversarial network (GAN). For example, U‐Net was utilized to estimate the local strain field in elastic composite materials.^[^ ^32^ ^]^ CNN was applied to predict the mechanical properties of fiber‐reinforced composites.^[^ ^33^, ^34^, ^35^ ^]^ Some researchers obtained dataset through numerical simulation based on the multi‐scale hydrogel fracture model, for prediction of the fracture behavior of hydrogel by CNN.^[^ ^36^, ^37^ ^]^ GAN, which trained two neural network models simultaneously, namely the generator and the discriminator,^[^ ^38^ ^]^ was implemented for composites, such as generating new structure with targeted mechanical properties,^[^ ^39^, ^40^ ^]^ predicting the microscale elastic strain field,^[^ ^33^ ^]^ and topology optimization.^[^ ^41^ ^]^

**Figure 1:**
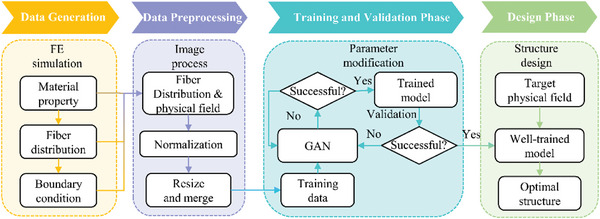
Workflow of DL‐based inverse structure design strategy.

In this paper, an inverse structure design strategy is proposed through a DL approach for meta‐fiber reinforced hydrogel composites with targeted mechanical field. The design process is schematically illustrated in **Figure** **1**, including four phases, i.e., data generation, data preprocessing, training and validation, and designing phases. In the data generation phase, the FE simulation for a meta‐fiber reinforced hydrogel composite is processed with chemical and temperature boundary conditions, to obtain the stress and displacement data after free‐swelling of the material. The fiber distribution image including the information of meta‐fiber, and the resulting image containing the information of stress and displacement field are normalized, and then the images are resized and proceeded to construct the dataset for DL model in the data pre‐processing phase. In the training and validation phases, a DL algorithm, GAN, is trained to design the fiber distribution by modification of the parameters between generator and discriminator. Finally, the fiber distribution of the composite is designed with targeted mechanical field in the design phase, based on the well‐trained GAN.

## 2 Methodology

In this section, a model for meta‐fiber reinforced hydrogel composite is first developed to simulate the free‐swelling progress by changing the temperature and external ionic concentrations, as described in Section 2.1. Subsequently, the fiber distributed structure and the mechanical field of the composite after free‐swelling are obtained by image process to the simulation results. In Section 2.3, the GAN is then trained based on the input data, stress or displacement field of composite, and output data, fiber distribution. The well‐trained GAN model is finally employed to design the optimized composite structure with targeted mechanical field.

### 2.1 Model Development for Meta‐Fiber Reinforced Hydrogel Composites

#### 2.1.1 Problem Statement

**Figure 2:**
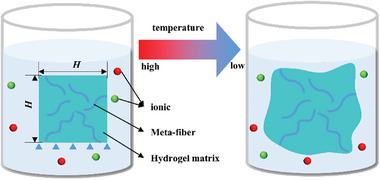
Swelling progress of hydrogel composite with chemical and temperature boundary conditions.

In this work, a 2D meta‐fiber reinforced hydrogel composite model is developed to simulate the free‐swelling process induced by change on temperature and external ionic concentrations of the external solution, based on theories of continuum mechanics. The schematic and computational zone are shown in **Figure** **2**, in which the square hydrogel matrix of length *H* is merged into solution with given temperature and ionic concentration, embedding a random number of continuous meta‐fiber with binary shapes, random distribution, and random orientation. The chemical and temperature boundary conditions are implemented on the whole composite boundary, and the fixed displacement boundary condition is set on the bottom boundary. Additionally, the meta‐fibers are cut into segments if they are across the composite boundary. Theoretically, several assumptions are made as follows: a) no overlap among meta‐fibers due to 2D assumption; b) infinite reaction time for fully swelling of the composite; c) perfect bounding between hydrogel matrix and meta‐fiber.

#### 2.1.2 Meta‐Fiber Structure

**Figure 3:**
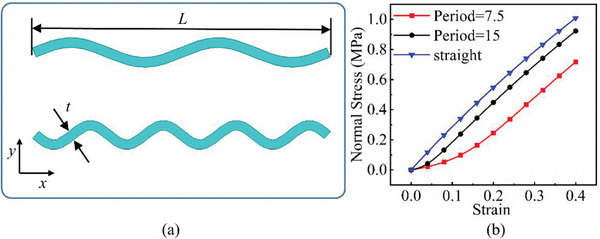
a) Geometry of meta fiber; b) comparison of stress‐strain curve between meta‐ and continuous fibers.

The hydrogel is reinforced by several meta‐fibers with sine structure, as sketched in **Figure** **3**a, instead of the traditional continuous fiber, in order to modify the stress distribution for a broad design space. The meta‐fiber is defined as an isotopically linear elastic solid with Poisson ratio ν and elastic modulus E. The overall height *H* and the width *t* of the meta‐fiber in free state are given same initial values among all the fibers. The shape of meta‐fiber is defined by a sine function, namely

(1) $$y=A\sin\left(\omega t+\varphi \right)$$

where *A*, *ω*, and φ are amplitude, angular frequency, and phase shift, respectively. Here, two types of the meta‐fiber are defined with different angular frequencies, such as ω_1_ = 3.75π and ω_2_ = 7.5π, which indicates the periods are 7.5 and 15.

The stress–strain analysis of two types of meta‐fiber with a tensile load is plotted in Figure 3b, comparing to the continuous fiber with same material parameters. Both types of meta‐fiber produce two phases of the deformation process, during which the fibers are first pulled straight and then stretched further. The transition behavior appears earlier in the meta‐fiber with a long period, than the one with a short period, as shown in the stress‐strain curve in Figure 3b. Additionally, the meta‐fiber with a short period suffers from a smaller load than the one with a long period, given the same displacement boundary condition. Therefore, the characteristic proves the meta‐fibsuffersesign space for mechanical property in a composite.

#### 2.1.3 Constitutive Equation of Hydrogel Matrix

The chain entanglements and functionality of junctions are considered to model the chemo‐mechanical coupling of hydrogel matrix. The swelling process is thus simulated by a hybrid free energy function^[^ ^17^ ^]^ involving both chemical and temperature fields, based on the Flory–Rehner free energy function. The total hybrid free energy density function is given by

(2) $$\begin{array}{ccc} \psi & = & -T\Delta S_{d}-T\Delta S_{s}+\Delta H_{mix}\\ & = & \psi_{el}^{c}+RT\left[\left(1-\frac{2}{f}\right)N_{c}\ln J+C_{s}\ln\frac{\upsilon_{s}C_{s}}{J}\right]\\ & & +\frac{1}{2}N_{s}RT\sum_{i=1}^{3}\left[\frac{\left(1+\eta \right)\lambda_{i}^{2}}{1+\eta \lambda_{i}^{2}}\right]+\frac{C_{s}}{J}RT\chi \end{array}$$

where *T* is the absolute temperature, Δ*S_d_* the entropy change of swelling process, Δ*S_s_* the elastic entropy, Δ*H*_mix_ the mixture enthalpy, $\psi_{\mathrm{el}}^{c}$ the elastic energy density function of the chemically cross‐linked networks, *R* the universal gas constant, *f* the functionality of the junctions between chains, *N_c_* the mole number of the chemically cross‐linked chains, *J* = λ_1_λ_2_λ_3_,λ_*i*_(*i* = 1, 2, 3) the principal stretches, $\upsilon_{s}$ the molar volume of solvent, *C_s_* mole number of solvent molecules, *N_s_* the mole number of the slip links, η the slippage parameter, and χ a dimensionless measure of the enthalpy of mixing, respectively.

The hybrid free energy density function is further converted to $\overset{\wedge }{\psi }=\psi -\mu C_{s}$ by Legendre transform can be written in terms of principal invariants.

(3) $$\begin{array}{ccc} & & \overset{\wedge }{\psi }\left(F,\mu ,T\right)=\frac{1}{2}N_{c}RT\\ & & \left\{I_{1}-3-\frac{4}{f}\log J+\frac{N_{s}}{N_{c}}\left[\begin{array}{c} \frac{\left(1+\eta \right)\left(I_{1}+2\eta I_{2}+3\eta^{2}J^{2}\right)}{1+\eta I_{1}+\eta^{2}I_{2}+\eta^{3}J^{2}}\\ +\log\left(1+\eta I_{1}+\eta^{2}I_{2}+\eta^{3}J^{2}\right) \end{array}\right]\right\}\\ & & -\frac{RT}{\upsilon_{s}}\left[\left(J-1\right)\log\left(\frac{J}{J-1}\right)-\frac{J-1}{J}\chi \right]-\frac{\mu }{\upsilon_{s}}\left(J-1\right) \end{array}$$

where **F** is the deformation gradient tensor, µ is the chemical potential, and *I_i_* (*i* = 1, 2, 3) is principal invariant, respectively.

The relationship between chemical potential µ_0_ and free stretch λ_0_ is then deduced by Equation (3), with a given initial temperature *T*_0_, chemical potential µ_0_, and free stretch λ_0_, written by

(4) $$\begin{array}{c} \frac{\mu_{0}}{RT\upsilon_{s}}=N_{c}\left(\lambda_{0}^{-1}-2\lambda_{0}^{-3}/f\right)+\frac{1}{\upsilon_{s}}\left(\log\left(1-\lambda_{0}^{-3}\right)+\lambda_{0}^{-3}+\lambda_{0}^{-6}\chi \right)\\ +N_{s}\left(1+\eta \right)\left[\begin{array}{c} \frac{\lambda_{0}^{-1}+4\eta \lambda_{0}+3\eta^{2}\lambda_{0}^{2}}{1+3\eta \lambda_{0}^{2}+3\eta^{2}\lambda_{0}^{4}+\eta^{3}\lambda_{0}^{6}}+\frac{\eta \lambda_{0}^{-1}+2\eta^{2}\lambda_{0}+\eta^{3}\lambda_{0}^{3}}{\left(1+\eta \right)\left(1+3\eta \lambda_{0}^{2}+3\eta^{2}\lambda_{0}^{4}+\eta^{3}\lambda_{0}^{6}\right)}\\ -\frac{\left(3\lambda_{0}^{2}+6\eta \lambda_{0}^{4}+3\eta^{2}\lambda_{0}^{6}\right)\left(\eta \lambda_{0}^{-1}+2\eta^{2}\lambda_{0}+\eta^{3}\lambda_{0}^{3}\right)}{{\left(1+3\eta \lambda_{0}^{2}+3\eta^{2}\lambda_{0}^{4}+\eta^{3}\lambda_{0}^{6}\right)}^{2}} \end{array}\right] \end{array}$$

The chemical potential between the hydrogel matrix and the solvent is equivalent at the initial state, in which the chemical potential in the solvent µ_*e*_ is approximated with ideal solution assumption by the following equation

(5) $$\mu_{e}\approx -RT\upsilon_{s}\sum_{\alpha =+,-}c_{e}^{\alpha }$$

where $\sum_{\alpha =+,-}c_{e}^{\alpha }$ is the sum of initial ionic species concentration.

Finally, the relationship between the stretch λ_0_ and ionic concentration $\sum_{\alpha =+,-}c_{e}^{\alpha }$ is derived by

(6) $$\begin{array}{c} \sum_{\alpha =+,-}c_{e}^{\alpha }=N_{c}\left(\lambda_{0}^{-1}-2\lambda_{0}^{-3}/f\right)+\frac{1}{\upsilon_{s}}\left(\log\left(1-\lambda_{0}^{-3}\right)+\lambda_{0}^{-3}+\lambda_{0}^{-6}\chi \right)\\ +N_{s}\left(1+\eta \right)\left[\begin{array}{c} \frac{\lambda_{0}^{-1}+4\eta \lambda_{0}+3\eta^{2}\lambda_{0}^{2}}{1+3\eta \lambda_{0}^{2}+3\eta^{2}\lambda_{0}^{4}+\eta^{3}\lambda_{0}^{6}}+\frac{\eta \lambda_{0}^{-1}+2\eta^{2}\lambda_{0}+\eta^{3}\lambda_{0}^{3}}{\left(1+\eta \right)\left(1+3\eta \lambda_{0}^{2}+3\eta^{2}\lambda_{0}^{4}+\eta^{3}\lambda_{0}^{6}\right)}\\ -\frac{\left(3\lambda_{0}^{2}+6\eta \lambda_{0}^{4}+3\eta^{2}\lambda_{0}^{6}\right)\left(\eta \lambda_{0}^{-1}+2\eta^{2}\lambda_{0}+\eta^{3}\lambda_{0}^{3}\right)}{{\left(1+3\eta \lambda_{0}^{2}+3\eta^{2}\lambda_{0}^{4}+\eta^{3}\lambda_{0}^{6}\right)}^{2}} \end{array}\right] \end{array}$$

The hydrogel matrix of the meta‐fiber reinforced hydrogel composite is modeled by the above hybrid free energy function, to characterize the deformation under the chemo‐mechanical coupling conditions, and numerically simulated by the commercial finite element software ABAQUS, via a user‐defined subroutine, hyperelastic material (UHYPER).

### 2.2 Image Preprocessing

The results from the FE simulation for the free‐swelling process of the composite are deposited by images, including the distribution of the meta‐fiber in the composite before swell and the distribution of the mechanical field of the composite after swell. The grey process is then implemented to reduce the dimension of the images, in which the color spectrum for field contours is proceeded by “pure white” [RGB = (255, 255, 255)] for upper bound and “pure black” [RGB = (0, 0, 0)] for the lower bound. As a result, the composite structure information and distribution of the stress are compressed into images with the size of 256×256 pixels.

The dataset including the input images, namely stress or displacement field after free‐swelling, and the output images, namely the corresponding fiber distribution, are normalized for the GAN training progress, as it may improve the accuracy and accelerate the convergence speed of the algorithm.^[^ ^42^ ^]^ During the pre‐processing stage, the images are normalized in the range [−1,1] by the Min‐max method, given by

(7) $$z_{i}=\frac{2x}{\max\left(x_{i}\right)}-1$$

where *z_i_* is the *i* ^th^ transformed data and *x*(*x*_1_,*x*_2_,⋅⋅⋅*x_n_*) are the original data.

### 2.3 Generative Adversarial Network

The complex relationship between meta‐fiber distribution and the mechanical field of meta‐fiber reinforced hydrogel composite after swelling is learnt by GAN based on the data set constructed in Section 2.2. GAN consists two components, generator *G*, and discriminator *D*. *G* is a U‐Net neural network to learn the mapping between the distribution of meta‐fiber and the composite mechanical field, and the convolutional neural network *D* distinguishes between the real image from the dataset and the predicted image produced the generator during the training process. The optimization problem of GAN is recognized as a minimax problem, in which the objective function of GAN is defined as

(8) $$\begin{array}{ccc} \min_{G}\max_{D}V\left(D,G\right) & = & E_{x∼p_{data}\left(x\right)}\left[\log D\left(x\right)\right]\\ & & +E_{z∼p_{z}\left(z\right)}\left[\log\left(1-D\left(G\left(z\right)\right)\right)\right] \end{array}$$

where *x* is sampled from the distribution of meta‐fiber, *p_data_(x)*, and *z* from the information of the stress field *p_z_(z)*. The generator *G* is trained to produce the excepted images, and the discriminator *D* distinguishes the produced images with the real images and returns the discrepancy to the generator. The generation and discrimination abilities of GAN is improved by the round‐and‐round generative and adversarial processes. The GAN algorithm reaches convergence if the generator and discriminator satisfy the Nash equilibrium state.

#### 2.3.1 Structure of Generator

**Figure 4:**
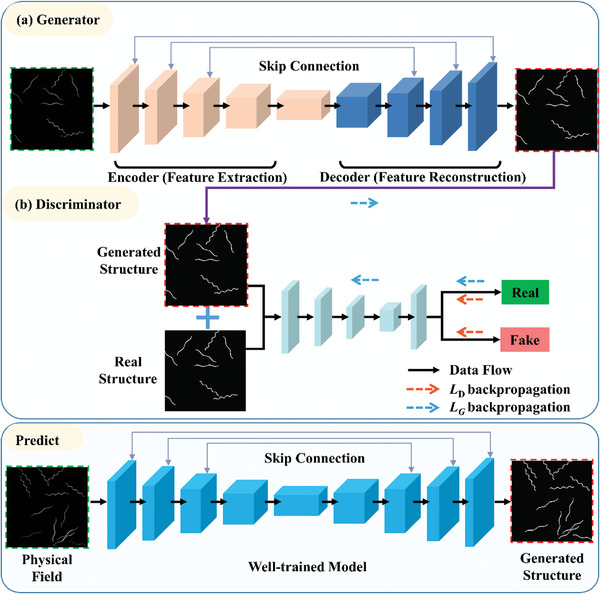
Structure of GAN consisting of generator and discriminator.

An encoder and decoder are involved in the Generator U‐Net with skip connections between mirrored layers. The encoder extracts the important feature from the input images, stress or displacement field after free‐swelling, to reduce the dimension by convolution kernels. The feature data is then proceeded to the decoder with the deconvolution calculation for feature reorganization to achieve the purpose of structure generation. The BatchNormalization is applied to avoid the over‐fit of the generator *G*. The complete architecture is shown in **Figure** **4**a. Approximate 31 000 000 parameters are trained in the generator and the loss function of *G* is defined as

(9) $$L_{gen}=L_{gan}+\lambda L_{l1}$$

where *L_gan_* is the loss between generated images and array zeros, *λ* = 100 is a constant coefficient, and *L_l1_* devotes the mean absolute error of generated and real images.

#### 2.3.2 Structure of Discriminator

The discriminator solves a two‐classification problem for identification of digitized image source from the generator or original dataset. A CNN acts as the domain function component in the discriminator, in which the first layer receives the real meta‐fiber distribution in the hydrogel composite from the dataset generated by FE simulation or predicted meta‐fiber distribution from the generator. The structure of the discriminator is schematically depicted in Figure 4b. Approximate 60000 parameters are trained in the discriminator and the loss function of *D* is defined as

(10) $$L_{dis}=L_{real}+L_{fake}$$

where *L_real_* calculate the loss between the normalized real image from dataset and identity matrix, and *L_fake_* is the loss between the fake image from generator and zero matrix.

In this work, the GAN model is trained to map the mechanical field with the distribution of meta‐fiber, for design of the meta‐fiber distribution in a hydrogel composite with a targeted mechanical field. The composite may be further produced by a 3D printing technique with dual jets.^[^ ^43^ ^]^

#### 2.3.3 Model Evaluation

The validation of the GAN is quantitatively evaluated by calculating the mean squared error (MSE) between a real meta‐fiber distribution and predicted distribution by GAN with the corresponding mechanical fields. Specifically, MSE calculates the mean square value between two images from pixel to pixel, given by

(11) $$\mathrm{MSE}=\frac{1}{M\times N}\sum_{0\leq i\leq N}\sum_{0\leq j\leq M}{\left(f_{\mathrm{ij}}^{\prime }-f_{\mathrm{ij}}\right)}^{2}$$

where *M* and *N* are the width and height of the images, respectively. 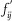 is the false image from the generator, and *f_ij_* the image from the dataset.

## 3 Validation, Result, and Discussion

In this section, the FE simulation of the hydrogel composite swelling is first configured in Section 3.1, following with the validation of hydrogel matrix swelling behavior, compared to the experimental results from the open literature.^[^ ^44^ ^]^ Subsequently, the training outcome and predicting performance of the GAN algorithm are presented in Section 3.2. The deep learning‐based inverse design strategy for meta‐fiber hydrogel composite is finally employed to design the composite structure with targeted mechanical field under representative working configuration, as displayed in Section 3.3.

### 3.1 Finite Element Simulation of Hydrogel Swelling

**Figure 5:**
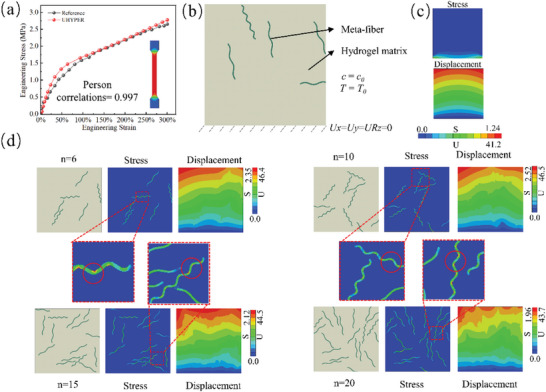
Parameter estimation of hydrogel matrix by FE simulation, and outcome mechanical field of meta‐fiber reinforced hydrogel after free‐swelling. a) Young's modulus estimation of the hydrogel matrix; b) the schematic of boundary conditions in FE simulation, where the dimensionless environmental concentration *c*_0_ = 0.03, the temperature *T*_0_ = 320K, and the displacement of the bottom of the composite is fixed; c) the swelling process of hydrogel matrix and the corresponding mechanical field; d) four representative simulation results of stress (unit: MPa) and displacement (unit: mm) fields of meta‐fiber reinforced hydrogel composite with the different number of meta‐fibers.

The numerical model for hydrogel matrix is validated comparing to the tensile experimental results of pure hydrogel materials from the open literature.^[^ ^44^ ^]^ The tensile process of a standard Dog‐bone specimen of hydrogel matrix is simulated through the commercial FE software ABAQUS with a user subroutine UHYPER based on the chemo‐mechanical coupling model^[^ ^17^ ^]^ presented in Section 2.1.3. The Person correlation coefficient between the numerical simulation and experimental results is 0.997, indicating a satisfactory agreement of user subroutine to quantitively describe the constitutive model of hydrogel matrix as shown in **Figure** **5**a.

In this work, the square meta‐fiber reinforced hydrogel composites experience a swelling process under constant chemical and thermal boundary conditions, in which the horizontal span of the fiber *L* is 30 mm, and the width 1 mm. In terms of the meta‐fibers, the isotopically elastic fiber is assumed with the 9 MPa Young's modulus and 0.3 Poisson ratio, and randomly distributed in the hydrogel matrix without overlap. With the purpose of generating recognizable results of stress or displacement field for the DL model, two different shapes of the meta‐fiber are designed here with 15 and 7.5 periods, and the total number of the meta‐fibers ranges from 5 to 20. Regarding the hydrogel matrix, the average Young's modulus is approximated by 900 kPa through a Dog‐bone model tensile test. Other initial simulation constants are listed in **Table** **1**.

**Table 1:** Input parameters of hydrogel in UHYPER subroutine.

*c*_0_	$\upsilon_{s}\left(N_{c}+N_{s}\right)$	*F*	*ϕ*	η	*T*_0_	χ_*L*_	χ_*H*_
0.03	0.001	4	0.5	0.2343	320K	0.1	0.7

The 100mm×100 mm composite is placed with a fixed boundary condition at bottom, as shown in Figure 5b. The swelling process of the hydrogel composite is driven by the temperature *T* dropping from 320 to 300K and a dimensionless environmental variable $\upsilon_{s}\sum_{\alpha =+,-}c_{e}^{\alpha }$ from 0.03 to 0.015. The advanced algorithm element CPE4H is employed for FE analysis, and the effectiveness of the simulation results for the swelling process are verified via testing the size and number of elements. The mechanical field of the swelled meta‐fiber reinforced hydrogel composite are then obtained by UHYPER in ABAQUS.

The simulation results for the swelling process of two representative meta‐fiber reinforced hydrogel composites are illustrated in Figure 5d. The stress concentration of the hydrogel composite is modified owing to the integrated meta‐fibers, comparing to those of the pure hydrogel materials as shown in Figure 5c. The stress concentration of the meta‐fiber reinforced hydrogel composite usually appears at concave side in the middle of the meta‐fiber, unlike the continuous fiber‐reinforced composite which produces the stress concentration at the end of fiber.^[^ ^45^ ^]^ The number of stress concentration regions relies on the shape of meta‐fiber, while the location of stress concentration regions is strongly associated with the meta‐fiber distribution, as shown in the stress nephogram of Figure 5d. Regarding the anisotropy displacement nephogram depicted in Figure 5d, the displacement at the two vertical sides of the meta‐fiber reinforced hydrogel composite has a large range from fixed point 0 to maximum 46.5 mm, approximating to 46% strain. Furthermore, the displacement at the opposite of the fixed side also presents obvious diversity among different meta‐fiber distributions in the hydrogel composite.

Based on the simulation results, the deformation behavior of the meta‐fiber reinforced hydrogel may be modulated by reasonably design of the meta‐fiber distribution. And the adjustment of stress field provides a wide range of design space on material function and structure designs. For example, the stress concentration of the composite, usually resulting in material failure, may be optimized by a property meta‐fiber distribution; the displacement field of the composite may be reorganized by the imported meta‐fiber to fit a desirable application.

### 3.2 Training Performance of GAN

The dataset for the GAN is composed by images from FE simulation for the free‐swelling of the meta‐fiber reinforced hydrogel composite with random fiber number and location. More particularly, a total number of 1800 image pairs, including the mechanical field distribution after swelling as the input images and the meta‐fiber distribution as the output images, are aggregated to train the deep learning model, 80% of which are randomly divided into training set and the left for validation set. The GAN is trained on the NVIDIA P2000 GPU with 10000 training iterations and 4 training sample in each loop. Furthermore, the generator in GAN uses the Adam optimizer with 4.0E‐4 learning rate, the decay of 1.0E‐8 and the discriminator applies the stochastic gradient descent optimizer with 4.0E‐4 learning rate without decay. The architecture is detailed in **Table** **2**.

**Table 2:** Parameters of each layer for GAN.

GAN	Layer Type	Output Shape
*G*	Input Layer	(256,256,1)
	Conv2D+BN+Relu+MaxPooling2D	(128,128,32)
	Conv2D+BN+Relu+MaxPooling2D	(64,64,64)
	Conv2D+BN+Relu+MaxPooling2D	(32,32,128)
	Conv2D+BN+Relu+MaxPooling2D	(16,16,256)
	Conv2D+BN+Relu+MaxPooling2D	(8,8,512)
	Conv2D+BN+Relu	(8,8,1024)
	Conv2D+UpSampling2D+ BN	(16,16,512)
	Conv2D+UpSampling2D+ BN	(32,32,256)
	Conv2D+UpSampling2D+ BN	(64,64,128)
	Conv2D+UpSampling2D+ BN	(128,128,64)
	Conv2D+UpSampling2D+ BN	(256,256,32)
	Conv2D + BN	(256,256,1)
	Output Layer	(256,256,1)
*D*	Input Layer	(256,256,1)
	Conv2D+Dropout+BN	(254,254,64)
	Conv2D+Dropout+BN	(126,126,32)
	Conv2D+Dropout+BN	(62,62,16)
	Conv2D+Dropout+BN	(30,30,16)
	Flatten	(None,14400)
	Dense	(None,1)
	Output Layer	(None,1)

**Figure 6:**
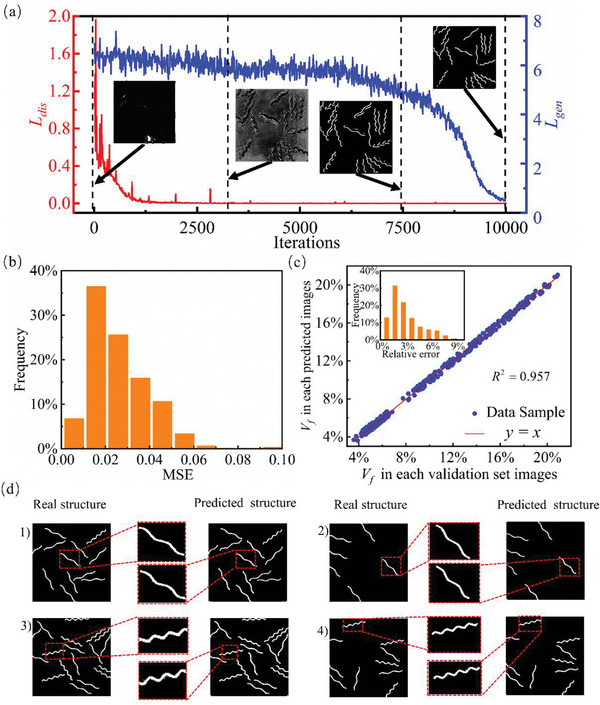
Deep learning model performance. a) loss function of GAN in training process; b) MSE of GAN between predicted structure and output images in validation dataset; c) regression analysis for fiber volume fraction *V_f_* of predicted structure; d) four representative examples for the structure predicted by stress field.

**Figure** **6**a shows the variation of the loss function in GAN with iterations from 0 to 10000. In this picture, the generator *G* and discriminator *D* are confronted continuously until the GAN model converges, in which the loss functions of the two components decrease with training iteration and are stable at a minimum value. Four representative predicted samples by the generator *G* are inset in Figure 6a at different iterations, presenting a more accurate process for the prediction of the composite, compared to the real structure in the validation set.

#### 3.2.1 Quantitative Analysis of GAN Based on the Stress Field

The frequency statistic of the prediction MSE by Equation (11) from pixel to pixel is presented by the bar chart in Figure 6b. All the predicted composite structure shows a relatively small prediction error as the entire MSE locates <0.1 while the majority <0.04. The regression is analyzed in Figure 6c for the fiber volume fraction *V_f_* defined by the fiber pixel over composite pixels in both predicted structure images by GAN, namely *x*‐axis in Figure 6c and the corresponding output images in validation dataset, namely *y*‐axis in Figure 6c. The inset figure shows the relative error distribution of GAN prediction, meaning that the relative error for the structure design is below 9%. The regression coefficient *R* ^2^ is 0.957 for the linear fitting to *y* = *x* between the predicted images and the validation output images, indicating that the structure of the prediction is highly correlated with the targeted structure. The comparisons between predicted structure and corresponding structure in validation set are illustrated in Figure 6d by four representative examples with two different meta‐fiber periods. As shown in this figure, the critical meta‐fiber information including the periodic, location, and orientation is accurately predicted by GAN. The meta‐fibers with the long period of 15 are perfectly predicted with smooth curves without extra pixels, as shown in the sub‐pictures 1) and 2) in Figure 6d. Regarding the mate‐fiber with the short period of 7.5, a few extra pixels are incorrectly predicted around each meta‐fiber, as shown in the sub‐pictures 3) and 4) in Figure 6d, but the vital features to reconstruct the composite structure are well predicted with a reasonable error, such as the smooth shape, period and location. Therefore, the well‐trained GAN model has a good accuracy in predicting the on‐demand structure of the meta‐fiber reinforcement hydrogel composite.

#### 3.2.2 Quantitative Analysis of GAN Based on the Displacement Field

**Figure 7:**
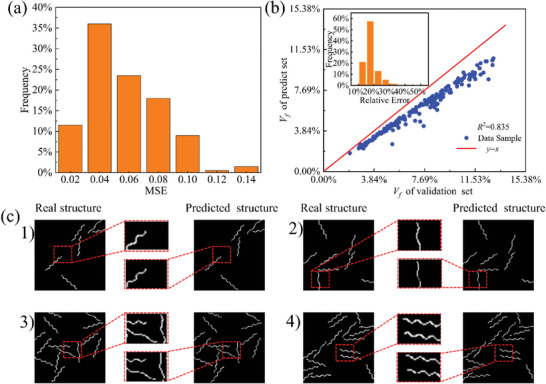
Deep learning model performance. a) MSE of GAN between predicted structure and output images in validation dataset; b) regression analysis for fiber volume fraction *V_f_* of predicted structure; c) four representative examples for the structure predicted by stress field.

Similar to the analysis of the prediction based on targeted stress, **Figure** **7** shows the results of the displacement field designed hydrogel composites. Figure 7a indicate that the predict structure highly similar to the real structure, and its relative error is <0.04, most of which are concentrated at 0.01. Figure 7b presents the analysis of the regression for the fiber volume fraction, which is defined as the ratio of fiber pixels to composite pixels in both the predicted structure images by GAN (*x*‐axis) based on the displacement field and the corresponding output images in the validation dataset (*y*‐axis). The inset figure displays the distribution of relative errors of GAN prediction, which reveals the relative error for structure design is below 17.5%. The regression coefficient *R* ^2^ between the predicted and validation output images is 0.835 by linear regression analysis, which shows the highly correlation, indicating that the predicted structure is closely related to the targeted structure.

The comparison of predicted structures with corresponding structures in the validation set is shown in Figure 7c. Four representative examples with two different meta‐fiber periods were used, and the results show that the GAN may accurately predict key information about meta‐fibers, including their period, position, and orientation. When designing fiber‐reinforced hydrogel structures using the displacement field, the GAN model may predict the position and period of fibers well in structures containing the lower *V_f_* of fibers. It is also found that the GAN model predicts the fiber structure with a good response to the period and position of the fibers as shown in Figure 7c (1) with an increase of fibers number, but some pixels in meta‐fibers are missed, as shown in Figure 7c (2). As a result, the prediction error of volume fraction of fiber *V_f_* based on displacement field is larger than that based on stress field. However, the discontinuous pixels in fibers are connected in the post‐process based on the physical reality of continuous materials, resulting in limited influence on the predicted composite structure as long as the key location parameter are well‐predicted, such as period, position, and orientation. It is worth mentioning that increasing the resolution of the input images or the depth of the GAN model may improve the predictive accuracy, resulting in an increase in computational cost as well. Regarding a structure generation task for composites, the reinforcement phase in the present work, namely meta‐fiber, has certain shape and parameter, as introduced in Section 2.1.3. In the other words, it is necessary to polish the generated composite structure in post‐processing, due to the manufacture practice, by smoothing the unreal curve while maintaining the important characteristics, such as fiber position, orientation and basic geometric parameters. Therefore, a balance between computational cost and absolute predictive accuracy is achieved in the present work.

### 3.3 Case Study I: Structure Design of Stress Field on Meta‐Fiber Reinforced Hydrogel Composite for Drug Delivery by GAN Method

One of the attractive applications of hydrogel composite is drug delivery, benefiting from the large deformation of hydrogel caused by absorbing surrounding biological liquid, as well as the sensitivity of environmental stimuli.^[^ ^4^, ^44^, ^46^ ^]^ As an efficient way of delivering the drug to a target destination in human body, the hydrogel carrier protects the drug from dissolving before reaching the focus and starts to release the resorted drug via a desired destruction encouraged by environmental stimuli.

In this section, a meta‐fiber reinforced hydrogel composite is framed by a GAN for drug delivery, through design of the fiber distribution to obtain a desired stress concentration and distribution of the overall composite. According to the simulation results presented by Figure 5d, the number and location of the stress concentration areas may be used as an important clue for structure design of the meta‐fiber reinforced hydrogel composite by arrangement of shape and number of the meta‐fibers, in order to predict the destruction position of the hydrogel carrier for drug delivery.

**Figure 8:**
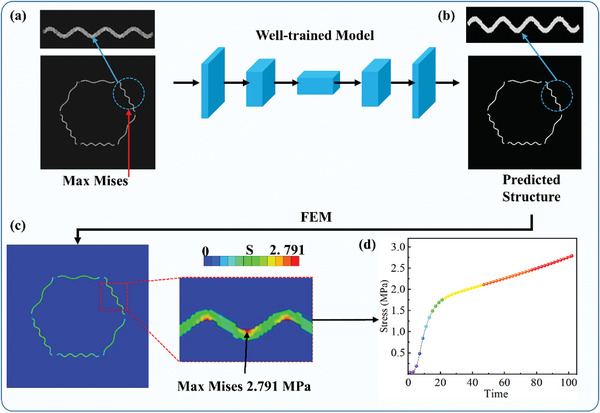
The workflow to predict the structure with targeted stress field and the changing processes of stress at the point of destructed position.

In this part, the meta‐fiber is set into a hexagonal shape as a reinforcing phase embedded in the hydrogel matrix to simulate multi‐point drug release problems. In **Figure** **8**a, a stress nephogram with the sine shape is considered to simulate the condition in which the drug is released from the center of the meta‐fiber reinforced hydrogel carrier. The initial stress field is set with the sine shape and the Max Mises value is around the meta‐fibers, and then the targeted stress field as the input for the GAN model to predict the structure. After the predicted structure is obtained by the GAN model, the structure depicted in Figure 8b is simulated by the FEM, and the Mises stress nephogram is shown in Figure 8c. The maximum stress in the predicted structure is found to be in the center of meta‐fiber with a maximum stress of 2.791 MPa after finite element calculations, and there are also stress concentration areas on the concave side of the meta‐fibers, which is consistent with the conclusion obtained in Section 3.1. Figure 8d shows the Mises stress changing process at the point of damage, and the stress values increase with the swelling time. Meanwhile, the rate of change in stress is sensitive to changes in the environment, indicating that the function of drug release in different environments may be achieved. In the same way, other fiber‐reinforced hydrogel carriers may be designed to achieve single and multi‐point drug release problems based on deep learning models as shown in **Table** **3**.

**Table 3:** Some representative examples for meta‐fiber reinforced hydrogel carrier.

	Example 1	Example 2	Example 3	Example 4
Stress field	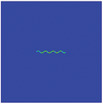	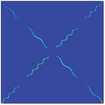	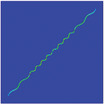	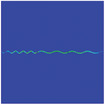
Real structure	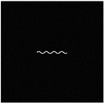	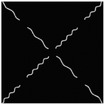	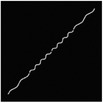	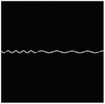
Predicted structure	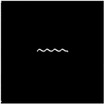	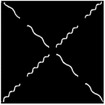	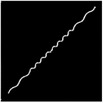	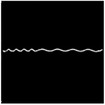
Relative error	7.15%	8.52%	3.83%	5.91%

### 3.4 Case Study II: Structure Design of Displacement Field on Meta‐Fiber Reinforced Hydrogel Composite for Flexible Sensors by GAN Method

Another remarkable application of hydrogel composites is to generate the special electronically signal for the anisotropic of deformation by controlling the fiber distribution during the swelling process. It has been extensively used as perceptive components in the fields of wearable strain sensors,^[^ ^47^, ^48^, ^49^ ^]^ bionic electronic skins,^[^ ^50^, ^51^ ^]^ and pressure sensors^[^ ^52^, ^53^, ^54^ ^]^ due to their excellent biocompatibility and adjustable deformation pattern. This method enables the hydrogel to sense the mechanical motion of the human body while monitoring physiological behaviors such as sweating, which is realized in the form of a tunable distribution of fibers after the hydrogel absorbs water to change the electrical resistance response characteristics of the hydrogel composite. In addition, the GAN model's directional structure design of fiber‐reinforced hydrogel composites under the targeted displacement field makes the hydrogel sensing performance highly tunable, which is potentially valuable in multi‐scenario adaptive interactions between humans and machines. Similar to the design strategy of the stress field, a targeted displacement field is employed to obtain the hydrogel composites with the distribution of meta‐fiber via GAN. On the basis of Figure 5d, structures with anisotropic displacement fields may be obtained by configuring the number of fibers and their distribution, the targeted displacement field is employed as a vital target for designing the meta‐fiber distribution of hydrogel composites to accomplish the aim of designing flexible sensor devices that meet the mechanical properties of the target with GAN models.

**Figure 9:**
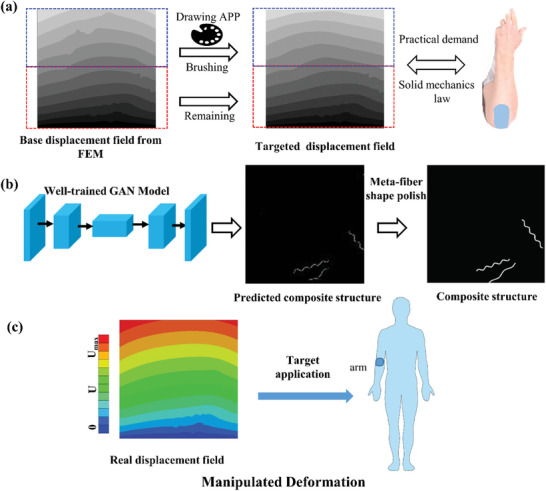
An example of workflow to predict composite structure with targeted displacement field; a) preparation of the targeted displacement field by combining the base displacement field from FE simulation (remaining the bottom half) and revised displacement field based on practical demand under the solid mechanics law (the up half), through the tools in drawing APP. b) generation of the composite structure by the well‐trained GAN model and shape polish for future manufacture, c) the contour of displacement field of designed hydrogel composite.

**Figure** **9** presents a general workflow to predict composite structure with targeted displacement field based on proposed GAN model. As shown in Figure 9a, the designer is suggested to selecting a displacement field image from the FE simulation dataset and keeping part of the image as a foundation. After that, the targeted displacement field is plotted by implementing brush tool of a drawing Application (APP) to adjust the grey contour band with a reference of practical demand of displacement field, under the prior knowledge of solid mechanics law. The prepared image of targeted displacement field is then input into the GAN model to predict the composite structure, as shown in Figure 9b. After meta‐fiber shape polishing, the composite structure is generated for the future manufacture. The FE simulation of the real displacement of the generated composite structure is illustrated in Figure 9c for verification, presenting a satisfactory result comparing to the targeted displacement field in Figure 9a. The designed meta‐fiber reinforced hydrogel targeted could be further applied for the applications on tissue engineering, such as targeted drug delivery and controlled release, flexible sensor, and artificial skin. Some representative examples for design of meta‐fiber reinforced hydrogel composites are presented in **Table** **4**.

**Table 4:** Some representative examples for meta‐fiber reinforced hydrogel composites.

	Example 1	Example 2	Example 3	Example 4
Displacement field	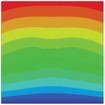	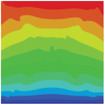	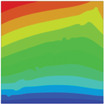	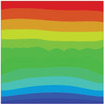
Real structure	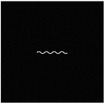	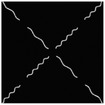	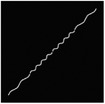	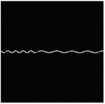
Predicted structure	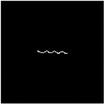	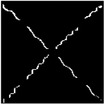	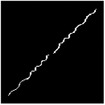	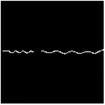
Relative error	5.47%	10.39%	7.34%	6.77%

It is also worth mentioning that the trained GAN model requires that the targeted strain or displacement field follows the basic solid mechanics laws, for example, the displacement at the bottom should remain zero and the contour line couldn't dramatically change, because all the data is collected under the same boundary condition. This issue indeed constrains the generalization capability of the current model, and may be solved by enlarging the dataset with other boundary conditions.

## 4 Conclusion

In this paper, a deep‐learning‐based structure design strategy is proposed to achieve a targeted mechanical property for meta‐fiber reinforced hydrogel based on a given mechanical field. A series of FE model are first developed for hydrogel composites reinforced by meta‐fibers with different numbers, sizes, orientations, and locations. The relationship between the mechanical field including (stress and displacement fields) and the corresponding meta‐fiber distribution in hydrogel composites is characterized by the deep learning algorithm GAN. As a result, the structure of the meta‐fiber reinforced hydrogel composite is predicted with a given stress field or displacement field for a targeted mechanical property by the well‐trained GAN. The conclusions are drawn as follows:

1. The meta‐fibers reinforce the mechanical properties of hydrogel composites. The integration of meta‐fiber into the hydrogel matrix induces the hydrogel to become anisotropic during the swelling process, in which both the period and distribution of meta‐fiber affect the mechanical field distribution of hydrogel composites after free‐swelling.
2. A GAN model is implemented to obtain an end‐to‐end mapping between the mechanic properties and meta‐fiber distributions. The GAN model predicts desired fiber distribution with a given mechanical field (including stress and displacement field) for a targeted mechanical property, while significantly reducing computational price.
3. The proposed GAN‐based structure design strategy is used for the design of the structure of drug release devices in a 2D‐material scenario. The reasonable structure of meta‐fiber reinforced hydrogel composite is designed based on the requirement of drug‐releasing process and the applications to monitor the health for human body. The method provides a new guiding approach and inspiration for the application of fiber‐reinforced hydrogel composites in the biomedical field.

## Conflict of Interest

The authors declare no conflict of interest.

## Data Availability

The data that support the findings of this study are available from the corresponding author upon reasonable request.

## References

[1] F. Zhang , L. Xiong , Y. Ai , Z. Liang , Q. Liang , Adv. Sci. 2018, 5, 1800450. 10.1002/advs.201800450 PMC6096994 30128253

[2] X. Le , W. Lu , J. Zhang , T. Chen , Adv. Sci. 2019, 6, 1801584. 10.1002/advs.201801584 PMC6402410 30886795

[3] C. Cui , T. Wu , X. Chen , Y. Liu , Y. Li , Z. Xu , C. Fan , W. Liu , Adv. Funct. Mater. 2020, 30, 2005869.

[4] Q. Liu , X. Ye , H. Wu , X. Zhang , Int. J. Mech. Sci. 2022, 215, 106963.

[5] Y. Zhu , Q. Zhang , X. Shi , D. Han , Adv. Mater. 2019, 31. 10.1002/adma.201804950 30815920

[6] Q. Peng , J. Chen , T. Wang , X. Peng , J. Liu , X. Wang , J. Wang , H. Zeng , InfoMat 2020, 2, 843.

[7] X. Du , H. Cui , B. Sun , J. Wang , Q. Zhao , K. Xia , T. Wu , M. S. Humayun , Adv. Mater. Technol. 2017, 2, 1700120.

[8] J. Liu , B. Yang , M. Li , J. Li , Y. Wan , Carbohydr. Polym. 2020, 227, 115335. 31590851 10.1016/j.carbpol.2019.115335

[9] H. Peng , X. Gao , K. Sun , X. Xie , G. Ma , X. Zhou , Z. Lei , Chem. Eng. J. 2021, 422, 130353.

[10] P. Lai , S. Yu , Polymers 2021, 13, 688. 33668913 10.3390/polym13050688 PMC7956583

[11] X. Lin , X. Zhao , C. Xu , L. Wang , Y. Xia , J. Polym. Sci. 2022, 60, 2525.

[12] B. Xue , Z. Bashir , Y. Guo , W. Yu , W. Sun , Y. Li , Y. Zhang , M. Qin , W. Wang , Y. Cao , Nat. Commun. 2023, 14, 2583. 37142590 10.1038/s41467-023-38280-4 PMC10160100

[13] J. Cheng , Z. Jia , T. Li , J. Mech. Phys. Solids 2020, 138, 103893. 10.1016/j.jmps.2020.103920 PMC7595329 33132418

[14] Y. Liu , H. Zhang , J. Wang , Y. Zheng , Int. J. Appl. Mech. 2017, 08, 1640003.

[15] Y. Liu , H. Zhang , J. Zhang , Y. Zheng , Eur. J. Mech. A/Solids 2015, 54, 171.

[16] S. Motiwale , M. D. Russell , O. Conroy , J. Carruth , M. Wancura , A. Robinson , E. Cosgriff‐Hernandez , M. S. Sacks , J. Mech. Behav. Biomed. Mater. 2021, 125, 104877. 34695661 10.1016/j.jmbbm.2021.104877 PMC8818123

[17] Q. Yang , L. Ma , J. Shang , Int. J. Solids Struct. 2013, 50, 2437.

[18] Q. Ma , H. Cheng , K.‐I. Jang , H. Luan , K.‐C. Hwang , J. A. Rogers , Y. Huang , Y. Zhang , J. Mech. Phys. Solids 2016, 90, 179. 27087704 10.1016/j.jmps.2016.02.012 PMC4831080

[19] X. Tan , S. Chen , B. Wang , J. Tang , L. Wang , S. Zhu , K. Yao , P. Xu , Extreme Mech. Lett. 2020, 41, 100990.

[20] K. K. Dudek , D. Attard , R. Gatt , J. N. Grima‐Cornish , J. N. Grima , Materials 2020, 13, 193. 32397654 10.3390/ma13092193 PMC7254355

[21] Q. Zhang , X. Xu , D. Lin , W. Chen , G. Xiong , Y. Yu , T. S. Fisher , H. Li , Adv. Mater. 2016, 28, 2229. 26788692 10.1002/adma.201505409

[22] G. Z. Zeng , R. L. Zu , D. L. Wu , W. X. Shi , J. F. Zhou , J. Y. Zhao , Z. W. Liu , H. M. Xie , S. Liu , Exp. Mech. 2021, 61, 1261.

[23] O. Ibragimova , A. Brahme , W. Muhammad , J. Lévesque , K. Inal , Int. J. Plast. 2021, 144, 103059.

[24] H. Wang , H. Qi , X. Ren , L. Tang , Q. Dong , KSCE J. Civil Eng. 2022, 26 , 248.

[25] G. Zhong , Y. Hou , W. Dou , Int. J. Mech. Sci. 2019, 153–154, 445.

[26] Q. Wang , Z. Wu , J. Huang , Z. Du , Y. Yue , D. Chen , D. Li , B. Su , Composites, Part B 2021, 223, 109116.

[27] B. Xu , K. Lee , W. Li , M. J. Yaszemski , L. Lu , Y. Yang , S. Wang , Mater. Des. 2021, 211, 110150.

[28] B. Yiming , T. Liu , G. Nian , Z. Han , Z. Jia , S. Qu , Extreme Mech. Lett. 2020, 35, 100643.

[29] K. Guo , Z. Yang , C. Yu , M. J. Buehler , Mater. Horiz. 2021, 8, 1153. 34821909 10.1039/d0mh01451f

[30] M. Zhang , Y. Jing , J. Zhang , Z. Sheng , Y. Hou , J. Xu , B. Chen , J. Liu , M. Wang , X. Hou , Interdiscip. Mater. 2022, 1, 157.

[31] A. Bhaduri , A. Gupta , L. Graham‐Brady , Composites, Part B 2022, 238, 109879.

[32] M. Raj , S. Thakre , R. K. Annabattula , A. K. Kanjarla , Integr. Mater. Manuf. Innov. 2021, 10, 444.

[33] Z. Yang , Y. C. Yabansu , D. Jha , W.‐K. Liao , A. N. Choudhary , S. R. Kalidindi , A. Agrawal , Acta Mater. 2019, 166, 335.

[34] E. Ford , K. Maneparambil , S. Rajan , N. Neithalath , Comput. Mater. Sci. 2021, 191, 110328.

[35] P. Bleiziffer , J. Hofmann , R. Zboray , T. Wiege , R. Herger , Eng. Appl. Artif. Intell. 2021, 104, 104351.

[36] S. Zheng , H. You , K. Y. Lam , H. Li , GIANT 2024, 17, 100242.

[37] S. Zheng , H. You , K. Y. Lam , H. Li , Adv. Theor. Simul. 2024, 2300776.

[38] I. Goodfellow , J. Pouget‐Abadie , M. Mirza , B. Xu , D. Warde‐Farley , S. Ozair , A. Courville , Y. Bengio , Adv. Neural Inf. Process. Syst. 2014, 3, 2672.

[39] Z. Yang , C. H. Yu , M. J. Buehler , Sci. Adv. 2021, 7, eabd7416. 33837076 10.1126/sciadv.abd7416 PMC8034856

[40] L. Qian , T. Yao , Z. Mo , J. Zhang , Y. Li , R. Zhang , N. Xu , Z. Li , Sci. Rep. 2021, 11, 21825. 34750453 10.1038/s41598-021-01307-1 PMC8575916

[41] J. Li , H. Ye , B. Yuan , N. Wei , Struct. Multidiscip. Optim. 2022, 65.

[42] O. Iruansi , M. Guadagnini , K. Pilakoutas , K. Neocleous , Int. J. Reliab. Saf. 2012, 6, 82.

[43] M. de Ruijter , A. Ribeiro , I. Dokter , M. Castilho , J. Malda , Adv. Healthcare Mater. 2019, 8, e1800418. 10.1002/adhm.201800418 PMC7116487 29911317

[44] Y. Gao , J. Peng , M. Zhou , Y. Yang , X. Wang , J. Wang , Y. Cao , W. Wang , D. Wu , J. Mater. Chem. B 2020, 8, 11010. 33188676 10.1039/d0tb02250k

[45] X. Peng , W. He , F. Xin , G. M. Genin , T. J. Lu , Int. J. Solids Struct. 2021, 222‐223, 111029.

[46] T. Kubota , Y. Kurashina , J. Zhao , K. Ando , H. Onoe , Mater. Des. 2021, 203, 109580.

[47] J. Song , S. Chen , L. Sun , Y. Guo , L. Zhang , S. Wang , H. Xuan , Q. Guan , Z. You , Adv. Mater. 2020, 32, 1906994. 10.1002/adma.201906994 31957099

[48] H. Zheng , M. Chen , Y. Sun , B. Zuo , Chem. Eng. J. 2022, 446, 136931.

[49] H. Liu , M. Li , S. Liu , P. Jia , X. Guo , S. Feng , T. J. Lu , H. Yang , F. Li , F. Xu , Mater. Horiz. 2020, 7, 203.

[50] Z. Zhang , Z. Chen , Y. Wang , Y. Zhao , Proc. Natl. Acad. Sci. USA 2020, 117, 18310. 32675247 10.1073/pnas.2007032117 PMC7414159

[51] H. Zhang , J. Guo , Y. Wang , L. Sun , Y. Zhao , Adv. Sci. 2021, 8, 2102156. 10.1002/advs.202102156 PMC8529447 34436831

[52] J. Huang , M. Zhao , Y. Cai , M. Zimniewska , D. Li , Q. Wei , Adv. Electron. Mater. 2020, 6, 1900934.

[53] M. Yin , Z. Yin , Y. Zhang , Q. Zheng , A. P. Zhang , Nano Energy 2019, 58, 96.

[54] F. Ye , M. Li , D. Ke , L. Wang , Y. Lu , Adv. Mater. Technol. 2019, 4, 1900346.

